# Head and neck cutaneous melanoma: 5-year survival analysis in a Serbian university center

**DOI:** 10.1186/s12957-020-02091-4

**Published:** 2020-11-29

**Authors:** Aleksandar Višnjić, Predrag Kovačević, Asen Veličkov, Mariola Stojanović, Stefan Mladenović

**Affiliations:** 1 Department of Social Medicine, Faculty of Medicine, University of Niš, Niš, 18000 Serbia; 2 Institute of Public Health of Niš, Niš, 18000 Serbia; 3 Department of Surgery, Faculty of Medicine, University of Niš, Niš, 18000 Serbia; 4 Clinic for Plastic and Reconstructive Surgery, Clinical Centre of Niš, Niš, 18000 Serbia; 5 Trauma and Reconstructive Surgery, Orthopedics, Kreisklinik Roth, Roth, 91154 Germany

**Keywords:** Melanoma, Head and neck, Survival, Predictors

## Abstract

**Background:**

Head and neck melanoma (HNM) is specific from the anatomical and etiopathogenetic aspects. In addition to morphopathological parameters, rich vascularization and lymphatic drainage of the head and neck affect the occurrence of lymphogenic and hematogenous metastases, as well as the metastases on both sides of the neck.

**Methods:**

A retrospective cross-sectional study included cutaneous melanoma patients who underwent surgery at a clinical center over a 10-year period. The clinical follow-up was at least 60 months. The Kaplan-Meier method was used for the survival analysis. The predictor effect of certain independent variables on a given dichotomous dependent variable (survival) was measured by the Cox regression analysis.

**Results:**

The analysis of demographic and clinical characteristics of 116 patients with HNM revealed that there was no statistically significant difference in age and gender in the total sample. Thirty-three (28.45%) patients were already in stage III or IV of the disease at the first examination, which affected the overall survival rate. The overall 5-year survival was 30.2%. No statistically significant difference in 5-year survival was found in relation to age and location. The period without melanoma progression decreased progressively in the advanced stage. Forty-nine patients (42%) underwent surgery for lymphogenic metastases in the parotid region and/or neck during the follow-up.

**Conclusions:**

Patients with HNM included in this study frequently presented an advanced stage of the disease at the first examination, which is reflected in a low rate of 5-year survival. Early diagnosis and adequate primary treatment can ensure longer survival.

## Background

Melanoma is one of the most malignant tumors in human population and the most malignant skin tumor. The incidence of melanoma varies around the world, and it has been steadily rising in recent decades. An increase of up to 200% over the last 20 years has been reported [1]. The increase in incidence is higher in males than in females [2].

Head and neck melanoma is specific in two aspects: anatomical and etiopathogenetic. In addition to local infiltration, which is assessed on the basis of a number of parameters (tumor thickness, number of mitoses, lymphovascular invasion, satellitosis), rich vascularization and lymphatic drainage of the head and neck affect the occurrence of lymphogenic and hematogenous metastases, as well as metastases on both sides of the neck in melanoma near the sagittal plane. The main prognostic factor is the thickness of the tumor. With the increase in the thickness of the tumor, the risk of developing lymphogenic metastases increases [3]. Metastases in the lymph nodes of the neck and parotid region, as well as lymph nodes in the occipital region for melanoma in the occipital region, are typical. The distribution of melanoma localization is as follows: face (52%), scalp (19%), neck (17%), ear (9%), and mucosal lesions (3%) [4]. The clinical course also depends on the localization of the primary tumor; therefore, the localization on the earlobe and scalp is mentioned as a risk factor for an aggressive clinical course. For head and neck melanoma, males are associated with a worse prognosis, while melanoma on the face mainly does not cause metastases [5, 6]. The etiology of head and neck melanoma is related to the exposure to UV radiation as an important factor. The head and neck area accounts for 9% of body surface area, while the prevalence of melanoma of these regions is higher [5, 6].

The median age at diagnosis was about 60 years, but a quarter of patients were younger than 45 [7]. Early diagnosis and adherence to established treatment guidelines significantly affect survival in these patients [8].

The American Joint Committee on Cancer (AJCC) assigns the following important factors: tumor thickness and ulceration for T category, the number of lymph nodes with micro or macro metastases, satellite infiltrations or in transit metastases for N category, and the presence of distant metastases and/or elevated plasma LDH levels for M category [9, 10].

Surgical treatment is the gold standard for treating cutaneous melanoma. Treatment of melanoma begins with an excisional biopsy. After the diagnosis is confirmed, the stage of the disease is determined. Then, a wide excision of the tumor is applied. Surgical margins of healthy tissue are defined as follows: for in situ melanoma 5 mm, for melanoma up to 1 mm it is 1 cm, and for the rest, the margin is 2 cm in the healthy tissue [11, 12]. The use of sentinel node biopsy is controversial. According to reports, it is performed in only 5–7% of all head and neck melanomas, and some studies show no difference in survival with or without sentinel biopsy [13–16]. Therapeutic dissections are performed when lymph node enlargement is clinically and radiologically confirmed. Dissection can be classified as radical neck dissection, modified radical neck dissection, or extended neck dissection. When clinically indicated, parotidectomy is performed in the form of a superficial parotidectomy, for melanoma in the parotid masseteric region and in front of a vertical line passing through the external auditory canal. In melanomas located in front of a vertical line passing through the external auditory canal, a dissection of the I–IV group of neck nodes is performed. For melanoma behind the vertical line through the external auditory canal, a posterolateral dissection is performed, which includes group V of the neck nodes and postauricular and suboccipital nodes [17]. In clinically positive metastases in the parotid region, some authors suggest elective neck dissection because they found the existence of occult metastases in 38% of patients [18].

Five-year survival for scalp and neck cutaneous melanoma has been on the rise recently (93.9% cases have been reported). However, in positive lymph nodes, survival is up to 83.1%, which is lower than in other anatomical regions. In relation to tumor thickness, for T1 disease-specific, 5-year survival (5-year DS) is 96.7% and for T4 it is 62.3% [19, 20].

The aim of this paper is to analyze the incidence of head and neck melanoma in the total population of cutaneous melanoma and determine the prognostic significance of gender, age, localization, and disease stage at a 5-year survival rate. The period without disease progression and the type of surgery during the follow-up are also shown.

## Methods

The retrospective cross-sectional study includes patients with cutaneous melanoma over a 10-year period who underwent surgery at a university clinical center in Niš, Serbia. Patients with melanoma of the head and neck were selected from the medical reports. Demographic characteristics (gender, age), localization of melanoma, and TNM classification according to the American Joint Committee on Cancer (TNM classifications for cutaneous melanoma, eighth edition) [9] were analyzed. The stage of the disease was defined during conciliar examination, and all patients were clinically monitored at the oncology advisory board for at least 60 months. It was determined whether lymphogenic or hematogenous metastases and melanoma-specific mortality occurred.

The study procedures were carried out in accordance with the Declaration of Helsinki, and approvals of the Ethical Committee of the Faculty of Medicine of the University of Niš.

### Statistical analysis

The following statistical parameters are presented by descriptive statistical analysis: arithmetic mean, standard deviation, coefficient of variation, absolute frequency (*n*), and structure index (%). The comparison of the frequency of occurrence of individual modalities of attribute features between groups was performed by Pearson χ^2^ test/Fisher exact test. The comparison of the mean values of the numerical features between the two independent groups of respondents was performed by Student’s *t* test or Mann-Whitney *U* test. To measure the correlation of certain traits, a Pearson correlation analysis was performed. The Kaplan-Meier method was used for survival analysis. The predictor effect of certain independent variables on a given dichotomous dependent variable (survival) was measured by Cox regression analysis.

Statistical analysis was performed using SPSS program version 18.0 (SPSS Inc., Chicago, IL, USA). The threshold for statistical significance was the level of statistical error less than 5% (*p* < 0.05). The results of the statistical analysis are presented in tables and graphs.

## Results

In the period from 2005 to 2015, a total of 774 patients with cutaneous melanoma were registered at the oncology advisory board of the University Clinical Center of Niš (Serbia), and 116 of them were diagnosed with head and neck cutaneous melanoma (14.99%). Demographic and clinical characteristics of patients with head and neck melanoma are given in Table 1.

**Table 1:** Demographic and clinical characteristics of patients

	Number	Mean age (years)	Std. deviation
Gender			
Male	67	61.78	13.62
Female	49	61.45	14.35
	Gender		
	Male	Female	Total
Age			
< 30	1	2	3
31–40	4	2	6
41–50	10	7	17
51–60	16	12	28
61–70	14	11	25
71–80	18	10	28
81 +	4	5	9
Total	67	49	116
	Male (*n*, %)	Female (*n*, %)	Total
Location			
Face	23 (34.3%)	27 (55.1%)	50 (43.1%)
Scalp	27 (40.3%)	5 (10.2%)	32 (27.6%)
Ear	6 (9,0%)	5 (10,2%)	11 (9,5%)
Neck	11 (16.4%)	12 (24.5%)	23 (19.8%)
Total	67 (100.0%)	49 (100.0%)	116 (100.0%)
	Male	Female	Total
Stage			
IA	6 (8.96%)	11(22.45%)	17 (14.66%)
IB	17 (25.37%)	11(22.45%)	28 (24.14%)
IIA	13 (19.40%)	9(18.37%)	22 (18.97%)
IIB	4 (5.97%)	3 (6.12%)	7 (6.03%)
IIC	4 (5.97%)	5 (10.20%)	9 (7.76%)
III	14 (20.90%)	8 (16.33%)	22 (18.97%)
IV	9 (13.43%)	2 (4.08%)	11 (9.48%)
Total	67 (100.00%)	49 (100.00%)	116 (100.00%)

The Student *t* test shows that there was no statistically significant difference in age and gender in the total sample (*p* = 0.9). Melanoma was most often diagnosed in persons aged 50 to 80 years. The most common localization was on the face. Unfortunately, a significantly large number had an advanced stage of the disease with already present lymphogenic and/or hematogenous metastases at the first examination (stages III and IV were diagnosed in 33 patients) (Table 1).

The distribution of the location in relation to gender differs significantly for tumors on the face and scalp. Namely, 40% of all melanomas in males is on the scalp, while 55% of melanomas in females is on the face (Pearson χ^2^ = 13.102, *p* = 0.004).

The overall 5-year survival is 30.2% (Table 2). It is statistically significantly higher in females (Mann-Whitney *U* test, *p* = 0.020). In our sample, female patients lived on average 9 months longer than male patients.

**Table 2:** Five-year survival by gender in total

60-month survival		Gender		Total
		M	F	
Died	*n* (%)	51 (76.1%)	30 (61.2%)	81 (69.8%)
Survived		16 (23.9%)	19 (38.8%)	35 (30.2%)
Total		67 (100.0%)	49 (100.0%)	116 (100.0%)

There is no statistically significant difference in 5-year survival in relation to age (*T* test, *p* = 0.204) and localization (χ^2^ test, *p* = 0.323), but survival is significantly shorter if lymphogenic or hematogenous metastases (stages III and IV) were reported at the first surgeon’s examination (χ^2^ test, *p* < 0.001) (Table 3).

**Table 3:** Disease stage and 5-year survival

Stage	Died *n* (%)	Survived *n* (%)	Total *n* (%)
IA	1 (1.2)	16 (45.7)	17 (14.7)
IB	16 (19.8)	12 (34.3)	28 (24.1)
IIA	16 (19.8)	6 (17.1)	22 (19.0)
IIB	6 (7.4)	1 (2.9)	7 (6.0)
IIC	9 (11.1)	0 (0)	9 (7.8)
III	22 (27.2)	0 (0)	22 (19.0)
IV	11 (13.6)	0 (0)	11 (9.5)
Total	81 (100.0)	35 (100.0)	116 (100.0)

The disease-free interval was calculated for a follow-up period of 60 months, regardless of the fact that in some patients the follow-up period was significantly longer (up to 150 months). The period without melanoma progression decreases progressively in the advanced stage, and the occurrence of parenchymal metastases is more frequent in later stages (Table 4). Based on the decision of the oncology advisory board, specific oncological therapy was determined for parenchymal metastases (29 patients).

**Table 4:** Disease stage and disease-free period

Stage	Number of patients	Mean DF (months)	Location of metastasis	Number of patients	Period until the occurrence of metastasis (months)
IA	17	57.58	N	1	19
IB	28	33.89	M	2	2–60
			N	19	
IIA	22	21.81	M	5	6–50
			N	13	
IIB	7	19	M	2	6–40
			N	5	
IIC	9	11	M	4	4–20
			N	5	
III	22	9	M	16	1–32
			N	6	
IV	11	0	N/A		N/A
Total	116	N/A	M	29	N/A
			N	49	

*DF* disease free, *M* parenchymal metastases, *N* lymphogenic metastases

Forty-nine patients (42%) underwent surgery for lymphogenic metastases in the parotid region and/or neck and superficial parotidectomy and radical neck dissection were performed (Table 5). In patients with stage III, extensive excision and radical neck dissection were simultaneously performed during the first surgery. During the follow-up of patients in stage III, six patients underwent neck surgery, including four reoperations on the same side of the neck, and two patients underwent dissection on the contralateral side of the neck.

**Table 5:** Disease stage and location of lymphogenic metastases

Stage	Number of patients	Location of lymphogenic metastases	Number of surgeries after initial treatment
IA	17	Neck	1
IB	28	Parotid	1
		Neck	13
		Parotid and neck	5
IIA	22	Parotid and neck	3
		Neck	10
IIB	7	Neck	5
IIC	9	Neck	5
III	22	Neck	6
IV	11	N/A	N/A
Total	116	Parotid	1
		Neck	40
		Parotid and neck	8

**Fig. 1:**
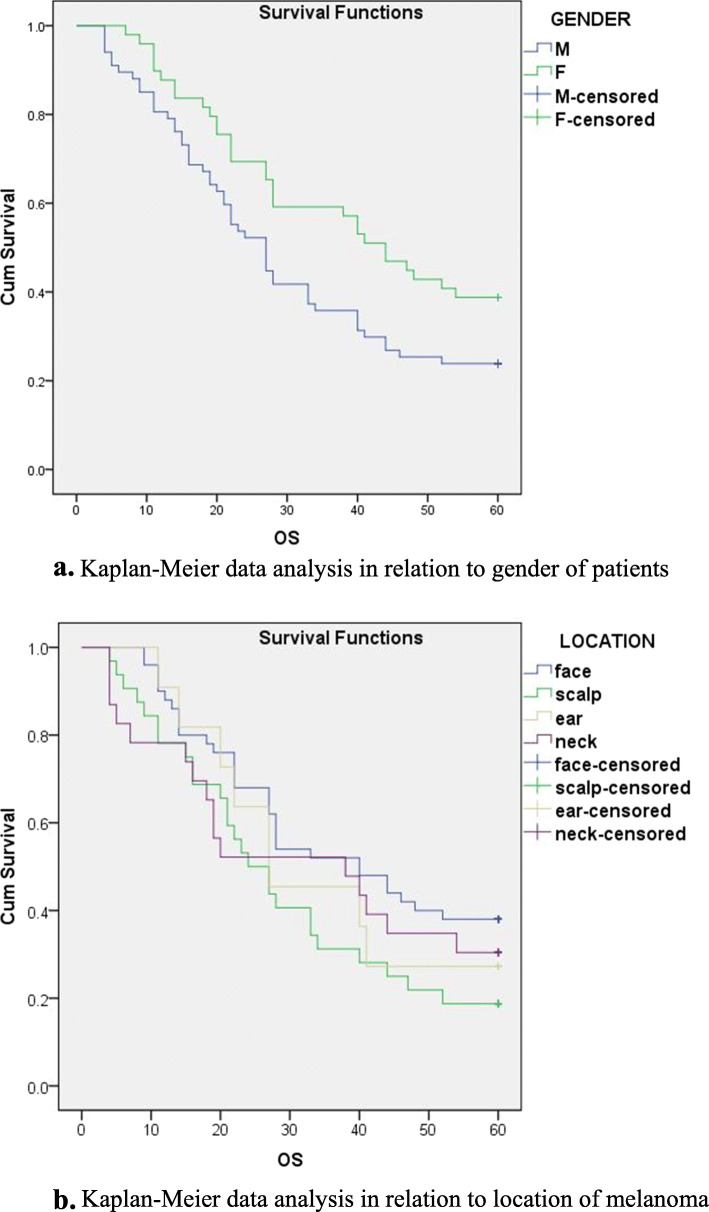
**a** Kaplan-Meier data analysis in relation to gender of patients. **b** Kaplan-Meier data analysis in relation to location of melanoma

Kaplan-Meier and Cox regression analysis were used to monitor the prognosis of patient survival in relation to the examined factors. Kaplan-Meier analysis (Fig. 1a) indicates a better prognosis in females (*p* = 0.031), while the localization of melanoma (Fig. 1b) does not affect survival (*p* = 0.253).

Cox regression analysis of survival predictors (backward stepwise method) indicates that the presence of metastases at the first examination, higher Breslow thickness, and higher number of nearby lymph nodes that have cancer (N) are predictors of poor survival. Size of the primary tumor (T), although seemingly with a pronounced prognostic value (HR = 1.524), is at the very limit of statistical significance (*p =* 0.047; 95% CI = 1.005–2.311), so it still cannot be interpreted as one of the predictors. Gender was also not a significant predictor of survival (Table 6). Localization of tumor, presence of distant metastasis (M), Clark level, and disease-free period (DFP) were as variables removed from the model at previous steps of the backward stepwise Cox regression analysis as non significant.

**Table 6:** Cox regression survival predictor analysis

Predictors	B	SE	Wald	df	*p*	HR	95.0% CI	
							Lower	Upper
M first ex	− 1.568	0.339	21.362	1	0.000	0.208	0.107	0.405
Breslow	− 0.832	0.287	11.232	1	0.005	0.375	0.245	0.672
T	0.421	0.212	3.931	1	0.047	1.524	1.005	2.311
N	− 0.372	0.142	6.891	1	0.009	0.689	0.522	0.910
Gender	0.424	0.252	2.835	1	0.092	1.528	0.933	2.502

*B* coefficient for the constant in the null model, *HR* hazard ratio, *M first ex* the presence of metastases at the first examination, *Breslow* Breslow depth, *T* size and extent of the main tumor, *N* degree of spread to regional lymph nodes (in TNM classification)

## Discussion

Our study included patients with head and neck melanoma (HNM) followed in the 5-year period from diagnosis. A total of 116 patients with HNM makes up 14.99% of all melanoma patients in the same time period, which represents a lower prevalence of HNM than in other studies [7]. HNM is more common in males (57.76%) and people older than 50 years (77.59%), but neither of the abovementioned predictors affects survival. El Sharouni et al. in a study of 54,645 patients point out to male gender as an important predictor of disease outcome [21]. The total 5-year survival in our sample is 30.2%, which is far less than the results in other studies [19, 20]. Reduced 5-year survival in our sample is associated with a large number of patients who consulted a doctor in an already advanced stage of the disease with present metastases (as many as 28.45% of patients in stages III and IV). Higher Breslow thickness, higher degree of tumor spread to regional lymph nodes (N), and the presence of metastases at the first examination were highlighted as predictors for the poor outcome. They significantly affect the progressive reduction of the disease-free period, which corresponds to the results of other authors [3, 7, 22].

Our study showed that the primary localization of melanoma did not affect 5-year survival. The results of other studies indicate that melanoma of the scalp has a more aggressive clinical course with more frequent local recurrences, shorter disease-free period, and almost twice the chance of disease progression [22–25]. The conclusions of other authors on the influence of scalp melanoma on 5-year survival are divided. According to some authors, these localisation of primary melanoma do not make a significant difference in 5-year survival compared to melanoma of other head and neck regions [25–28], while Ozao-Choy et al. indicate that melanoma of the scalp significantly adversely affects 5-year survival compared to melanoma of other localizations of the head and neck [29, 30].

Den Hondt et al. in their paper point out the importance of elective neck dissection in patients with metastases in the parotid region. In our sample, 18% of patients with metastases had metastases in the parotid region of which as many as 89% of patients had failure in cervical nodes. Elective neck dissection not only reduces failure rates in cervical nodes but also provides more accurate information about the stage of the disease and prognosis [31].

Radical neck dissection (RND) was performed in 70 patients with clinically and ultrasonographically proven positive lymph nodes. In 22 patients who were in stage III at the first examination, RND was performed in the same act as radical excision of cutaneous melanoma. In other 48 patients, RND was performed during a follow-up, four of whom underwent redissection. The literature shows a great range in recurrence rates after neck dissection (from 0 to 43%) [16, 32–37]. In a study with a similar number of patients, Andersen et al. [38] stated that the recurrence rate after RND was 11%, which is similar to the percentage in our sample (6.06%). One interesting fact from our sample is that recurrences after RND occurred only in patients who were in stage III at the first examination, while in patients who underwent RND during the follow-up, there were no recurrences.

Song et al. indicate the importance of using systemic therapy in patients who were in the third stage of the disease at the time of the first examination due to the fact that recurrences in the lymph nodes occur more often in these patients and they have a poorer prognosis. The use of systemic therapy in these patients significantly improves 5-year survival [39].

However, since the incidence of head and neck cutaneous melanoma continues to increase all over the world, and that it dramatically affects quality of life both in patients and caregivers, the primary prevention and early diagnosis should become a priority [40, 41]. Excessive sun exposure and overuse of solarium have been established as the major cause of cutaneous melanoma, so the effect of health education on this behavior could be valuable, along with the secondary prevention measures of particularly at risk populations [42–44].

## Conclusions

Patients with head and neck melanoma included in this study very often presented melanoma in an advanced stage of the disease at the first examination, which caused the low rate of 5-year survival. Treatment of head and neck melanoma is a great challenge due to frequent metastases to lymph nodes and the development of distant metastases. Therefore, in addition to adequate oncosurgical treatment, it is necessary to pay attention to postoperative monitoring and further treatment in accordance with treatment protocols.

## Data Availability

The datasets used and analyzed during the current study are available from the corresponding author on reasonable request.
